# Paediatric Visceral Leishmaniasis in Italy: a ‘One Health’ approach is needed

**DOI:** 10.1186/1756-3305-6-123

**Published:** 2013-04-29

**Authors:** Vincenzo Lorusso, Filipe Dantas-Torres, Francesco Caprio, Mariano Manzionna, Nicola Santoro, Gad Baneth, Domenico Otranto

**Affiliations:** 1 Department of Veterinary Medicine, University of Bari, Valenzano, Bari 70010, Italy; 2 Division of Pathway Medicine, University of Edinburgh, The Chancellor’s Building, 49 Little France Crescent, Edinburgh EH16 4SB, UK; 3 Department of Immunology, Aggeu Magalhães Research Centre, Oswaldo Cruz Foundation, Recife, Pernambuco, Brazil; 4 Department of Paediatrics and Neonatology, Ospedale ‘San Giacomo’, Monopoli, Bari, Italy; 5 Department of Biomedicine of the Evolutive Age, Azienda Ospedaliera Universitaria Policlinico, Bari, Italy; 6 School of Veterinary Medicine, Hebrew University, Rehovot, Israel

## Abstract

Here we describe a case of paediatric visceral leishmaniasis recorded in an infant initially suspected for acute lymphoblastic leukaemia due to the clinical and haematological presentation. Eventually the patient was found positive for *Leishmania infantum* infection and successfully treated. This case emphasises how pivotal a ‘One Health’ approach is for diagnosing this zoonotic disease; highlighting the importance of including Visceral Leishmaniasis in the differential diagnosis of leukaemia-like syndromes in infants travelling to, and living in, the Mediterranean region.

## Letter

Visceral leishmaniasis (VL) is an endemic zoonosis in the Mediterranean basin, where it is caused by the kinetoplastid protozoan *Leishmania infantum*, and transmitted by sand flies of the genus *Phlebotomus*[1]. After peaking in the post-second world war and, the incidence of infantile VL in Italy dramatically decreased to a few cases per year [2]. At present, however, the actual occurrence of the disease in the country seems to be underestimated, due to misdiagnosing and underreporting.

Here we point out the case of a 31-month-old Italian infant initially suspected for acute lymphoblastic leukaemia (ALL), due to his clinical and haematological presentation, and eventually found positive and treated for *L. infantum* infection.

**Figure 1:**
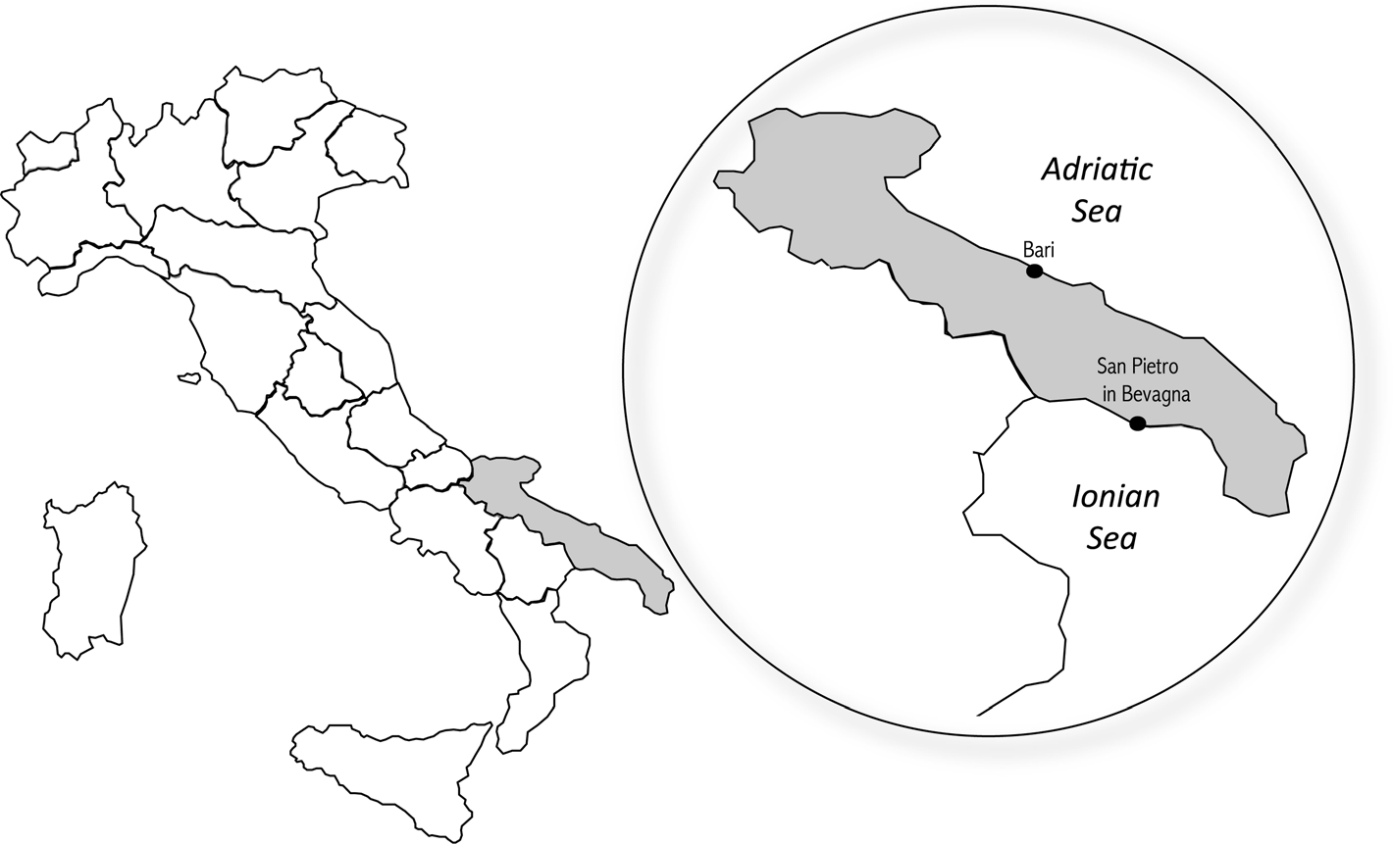
Map of Italy with focus on the Apulia region (grey) and localities where the child resided (black dots).

In August 2009, the child was taken to the emergency ward of the local hospital in Nardò (Lecce province, Apulia region) due to persistent hyperthermia (i.e., 39-41°C) accompanied by anorexia. He had been treated with paracetamol (250 mg/every six hours) for the previous seven days without any improvement. From the day of his birth until end July 2009, the infant had been healthily living in the suburbs of Bari (41°13′N, 16°87′E), the capital of the south-eastern Apulia region; however, during the four weeks prior to hospitalization, he had resided at a summerhouse in the coastal locality of San Pietro in Bevagna (40°30′N, 17°64′E, Brindisi province), on the Ionian Sea (Figure 1). At the initial check-up, the patient (15 kg of weight) presented with fever (38.7°C) and splenomegaly (spleen measuring 12.1 × 4.7 cm), and was therefore subjected to haematological examinations including blood count and serum protein electrophoresis (SPE). Laboratory results were indicative of anaemia, thrombocytopaenia, lymphocytosis and neutropaenia, hypoalbuminemia, and polyclonal (alpha-1 and gamma-type) hypergammaglobulinemia. High activities of serum aspartate transaminase (AST) and C-reactive protein were also recorded. Serological tests for the detection of Epstein-Barr and cytomegalovirus infections were negative. As his condition was thought to be attributable to a haematopoietic disorder, the next day the boy was referred to the paediatric ward at the hospital in Monopoli (Bari province), where he was subjected to further haematological testing. Laboratory studies showed microcytic anaemia accompanied by a more pronounced leukopaenia yet with lymphocytosis, thrombocytopaenia, hypoalbuminemia, and high AST activity. A suspected diagnosis of ALL was then considered, and the infant was referred to the paediatric oncohaematology ward at the ‘Policlinico’ University Hospital of Bari, while presenting with fever (40°C), palpable liver, and enlarged spleen reaching the umbilical transversa. A bone marrow aspirate from the sternum was obtained and no evidence of any leukaemic form was cytologically detected either at the University of Bari, or at the National Reference Center for infantile leukaemia in Padua, Italy. It was then that a suspicion of visceral leishmaniasis (VL) was raised, following the anamnestic information provided by the patient’s father, who practices as a veterinarian in the same region, known for being highly endemic for canine leishmaniasis [3]. The child’s parent recalled being bitten by numerous sand flies during his stay at the coastal locality in the former weeks. Several laboratory examinations were therefore carried out, including the serological rK39 and the indirect fluorescent antibody test (IFAT). In addition, a bone marrow biopsy was also cytologically examined for the detection of *L. infantum* amastigotes in macrophages. Both rK39 and cytological tests resulted negative. However, the patient was found positive by IFAT with an antibody titer of 1:640 (threshold of 1:40). A real-time PCR was carried out on a bone marrow sample that had initially tested as negative at cytology, giving positive results. As soon as the VL suspicion was confirmed, the child was successfully treated with an intravenous administration of liposomal amphotericin B (3 mg/kg/day) for five consecutive days (i.e., August 31-September 4) and on day +10 (i.e., September 9).

The present case highlights the importance of including VL in the differential diagnosis of leukaemia-like syndromes (e.g., splenomegaly and haematopoietic alterations) in infants living in or travelling to endemic areas like the Mediterranean basin. In this case, collecting information on the summer stay of the child at a seaside locality, apparently one of the commonest habits of middle class families in this part of Italy, eventually turned out to be pivotal anamnestic data, leading physicians to the correct interpretation of clinical and laboratory findings. In Italy, as well as in other southern European countries, the majority of patients diagnosed and treated for VL are indeed children aging under or equal to 3 years [4-6], due to the immaturity of their immune system [7]. Noteworthy, the name ‘*infantum*’ was initially attributed to this *Leishmania* species for causing a visceral syndrome predominantly in infants in the Mediterranean region.

As the number of contacts with the phlebotomine vector has been suggested as being related to the likelihood for the human host to develop clinical illness [7], it was suspected that the patient was infected during his stay at the seaside, when he was more likely exposed to insect bites due to the frequent outdoor activities undertaken by his family. This assumption is further supported by the high abundance of sand flies and stray dogs in the area, with high prevalence (~50%) of infected, and often asymptomatic, animals [3]. Furthermore, the abundance of other suitable reservoir host species, such as red foxes (*Vulpes vulpes*), enhances the risk of transmission of VL to humans in the surroundings [8]. Importantly, in a recent study, *Phlebotomus perniciosus* and *Phlebotomus neglectus*, both competent vectors of *L. infantum*[9], were found in the same region, with the highest abundance recorded in July and August [10]. This explains the common onset of the disease during the summer, as reported in other studies carried out in the Mediterranean region [4-6].

In this case, the conclusive diagnosis of VL was achieved by IFAT, already proven to be highly sensitive (i.e., 87–100%) and suitable for diagnosing VL in paediatric patients in presence of a strong clinical suspicion [11]. The false negative result achieved by cytology of bone marrow can be attributed to the lower sensitivity (i.e., 52–80%) of such a diagnostic approach especially at the early stage of infection [11]. The immunochromatographic rK39 test resulted negative, thus with low sensitivity, as previously reported in a field study in Sudan [11].

This case illustrates how a ‘One Health’ approach, based on the increased level of communication between physicians and veterinarians, is crucial for the management of VL in endemic areas [12]. In this scenario, veterinarians and public health authorities play an essential role in reducing the risk of infections in humans [3]. Where the elimination of the infection in the canine reservoir cannot be achieved, prompt diagnosis and treatment of human patients are indeed essential in order to reduce the public health impact of VL.

## Competing interests

The authors declare they have no competing interests.

## Authors’ contributions

VL and DO conceived this letter. FC collected clinical records and laboratory results. VL analysed clinical and laboratory findings, designed the first draft of the manuscript. MM and NS actively participated to the diagnostic investigation of the case, being the heads of the Oncohaematology and Paediatric wards of the Azienda Ospedaliera Universitaria ‘Policlinico’, Bari, and ‘San Giacomo’ Hospital in Monopoli, Bari, respectively. VL, FDT, FC, GB, and DO wrote the letter. All authors contributed to the critical interpretation of results and read and approved the final version of the manuscript.
